# A case of Paget-Schroetter syndrome (PSS) in a young judo tutor: a case report

**DOI:** 10.1186/s13256-016-0848-0

**Published:** 2016-03-18

**Authors:** Ruth Ijaopo, Victor Oguntolu, Dominick DCosta, Andrew Garnham, Simon Hobbs

**Affiliations:** New Cross Hospital, Royal Wolverhampton NHS Hospital, Heath Town, Wolverhampton, UK

**Keywords:** Paget-Schroetter syndrome, Catheter-directed thrombolysis, Deep vein thrombosis

## Abstract

**Background:**

We present a case of unsuspected Paget-Schroetter syndrome (also called *effort thrombosis*), one of the rare causes of upper extremity deep vein thrombosis. To the best of our knowledge, this was the first such case in our hospital. Our patient may have been discharged with an incomplete diagnosis and inappropriate management but for one of the few physicians who knew about effort thrombosis, made the diagnosis, and referred the patient promptly to the appropriate team.

**Case presentation:**

A 37-year-old Caucasian man who was an active judo tutor presented to the acute medical unit in our hospital. He had initially presented to his primary care physician complaining of redness and swelling of his right arm and elbow of 1 week’s duration. He had been prescribed some antibiotics by his general practitioner, but his symptoms worsened. At that point, he was referred to our hospital for further review of his arm swelling.

**Conclusions:**

When considering a diagnosis of deep vein thrombosis, the age, hobbies, and occupation, among other things, of individual patients should be put into context at all times to avoid missing rare causes such as Paget-Schroetter syndrome. This report is intended to raise awareness of this rare condition. Knowledge of this condition and its management is essential for all medical practitioners, especially medical doctors who are involved in unselected medical admissions in accident and emergency or medical admission units, where the majority of such patients are seen.

## Background

There has recently been a sharp rise in the incidence of upper extremity deep vein thrombosis (UEDVT), which currently accounts for about 10 % (annual incidence of 0.4–1 cases per 10,000 population) of all deep vein thrombosis (DVT) cases [1]. UEDVT commonly affects the axillary and subclavian veins. Paget-Schroetter syndrome (PSS), also called *effort thrombosis*, is an unusual cause of UEDVT and has remained mostly undiagnosed or misdiagnosed, probably due to lack of awareness of the syndrome. It is a rare condition, with an incidence rate of 1–2 per 100,000 population [2]; however, if left untreated, it can lead to significant deformity and morbidity [1]. Therefore, a high index of suspicion and thorough knowledge are necessary, especially among clinicians working in accident and emergency, medical admission, and clinical decision units.

## Case presentation

A 37-year-old, active Caucasian man was admitted to our hospital’s acute medical unit with a 1-week history of erythematous painful swelling of his right elbow and arm. He had no history of fever and rash. His systemic review was generally unremarkable. He worked as an engineer but also did judo tutoring in the evenings. He was normally fit and well, had no family history of thrombosis, and had not undergone surgery recently or in the past. He was not on any regular medication, denied any current or previous history of recreational drug use, and had no known history of drug allergy.

After a routine judo training session, he noticed acute onset of a red, swollen, and painful right arm. He presented to his general practitioner (GP), who made a diagnosis of cellulitis with possible trauma-related musculoskeletal injury. He was subsequently commenced on analgesics and antibiotics. About 1 week later, he attended a follow-up examination with his GP, who immediately referred him to the hospital because he showed no improvement. On admission, his temperature was 37.3 °C, his blood pressure was 124/74 mmHg, his pulse rate was 80 beats/minute, his respiratory rate was 16 breaths/minute, and his oxygen saturation was 96 % on room air.

His physical examination revealed noticeable swelling and redness from his right elbow to his shoulder as well as mild erythema and tenderness of the affected area. No superficially engorged vein was noted on his chest. All of his systemic examinations were essentially within normal limits. All of his blood workup results, including the coagulation profile, were unremarkable, except for a slightly elevated C-reactive protein level of 26 mg/l (normal range <5 mg/l).

The working differential diagnoses of possible olecranon bursitis and right arm cellulitis to rule out DVT were made. The patient was then commenced on parenteral antibiotics and low molecular weight heparin pending further investigations. The x-rays of his right elbow, right arm, and chest showed no abnormalities; however, venous Doppler ultrasonography revealed a right axillary DVT with extension into the cephalic and brachial veins.

Thrombophilia screening was performed, and the patient was started on warfarin with a plan to discharge him to home with community-based anticoagulant clinic follow-up. While waiting for discharge, he was moved to a medical outlier ward on the diabetes ward due to bed pressures. The consultant on the ward reviewed the patient’s case and made a diagnosis of PSS, and an urgent requests for consultation was sent to the vascular surgical team for review of further management.

**Fig. 1:**
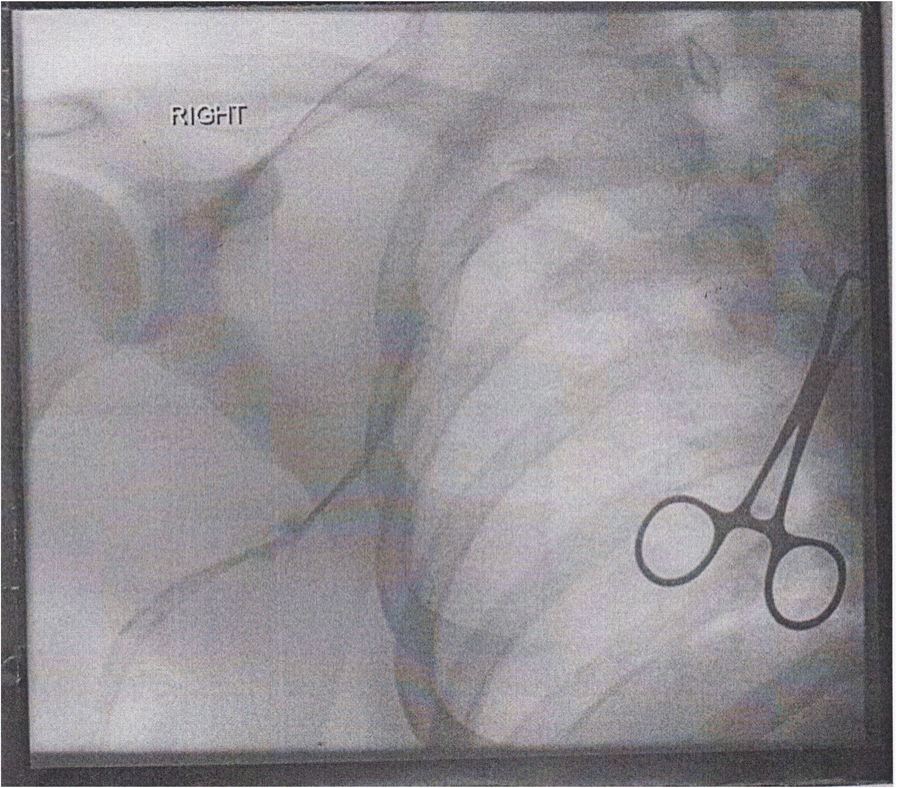
Prethrombolysis venogram

**Fig. 2:**
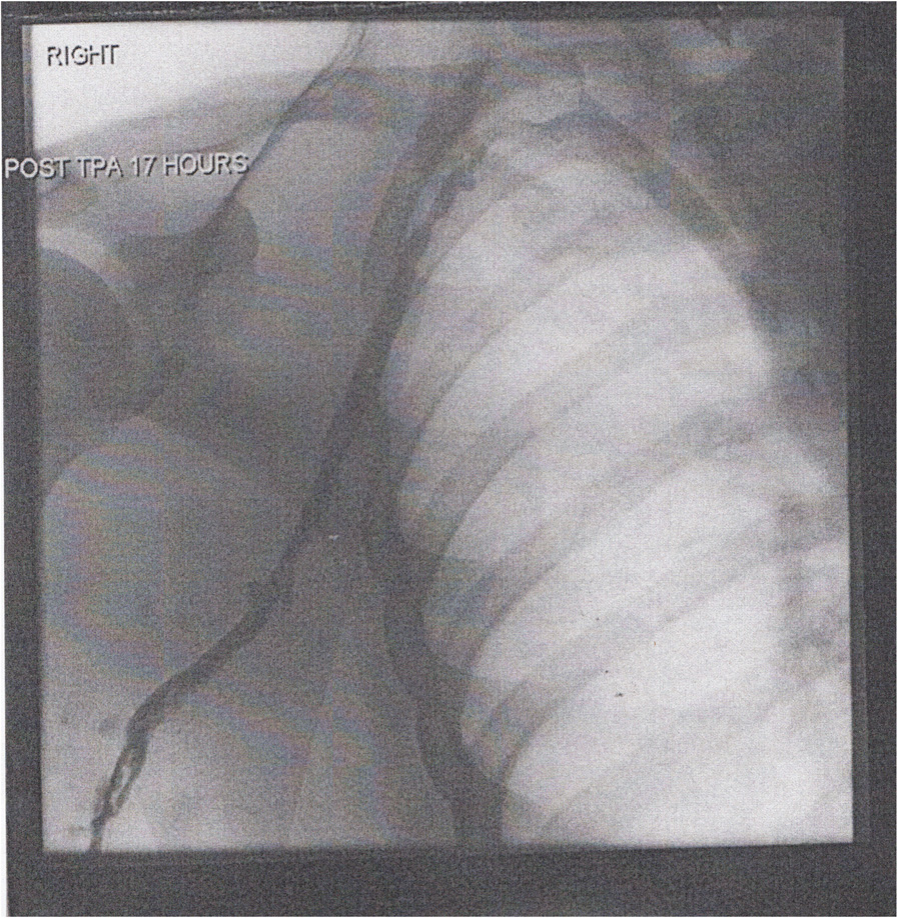
Postthrombosis venogram

The vascular team organized catheter-directed thrombolysis (CDT) to clear the patients’ extensive thrombosis. A prethrombolysis venogram (Fig. 1) with extensive thrombosis showed marked reduction in thrombus load within the axillary and subclavian veins after a postlysis venogram (Fig. 2) was performed. Following thrombolysis, an early thoracic outlet decompression via a transaxillary first rib resection was undertaken to reduce the risk of recurrent thrombosis and long-term morbidity. The BMJ Best Practice treatment guideline for thoracic outlet syndrome [3] provides a good illustration of this procedure. The patient had an uneventful postoperative recovery. He was subsequently anticoagulated and was discharged to home with a target international normalized ratio of 2–3. A few weeks after his surgical procedure, the patient was followed in the outpatient clinic. The swelling of his right arm had completely almost resolved. The patient reported no postoperative complications.

## Discussion

UEDVT is classified into primary and secondary cases, with the latter accounting for about 80 %. This is directly related to the increasing use of central venous catheters for chemotherapy, bone marrow transplantation, dialysis, pacemaker insertion, and parenteral nutrition. Other secondary causes are hypercoagulable state, use of oral contraceptive pills, and surgery involving the upper arm [4]. These groups of patients are best treated with anticoagulation alone. The primary cause, which accounts for the remaining 20 % of cases, is anatomical anomalies, either acquired or congenital; hence the term *effort thrombosis*, or PSS.

PSS was first described by Paget in 1875 and independently by Schroetter in 1884 [5]. It usually occurs in young, active, healthy, athletic people. The incidence is greater in males than females, and the mean age of occurrence is 30 years. The hallmark of PSS is a lifestyle involving vigorous, repetitive upper limb movement [1], especially in competitive swimmers, weightlifters, and javelin throwers, all of whom develop their shoulder girdle muscles to improve their sports performance.

The simple pathogenesis of PSS is that heavy exertion causes microtrauma to the vessel intima and leads to activation of the coagulation cascade [2]. A further explanation is that the repetitive movement of the upper extremities results in scalene muscle hypertrophy, in particular the subclavius muscle. This results in compression of the subclavian vein between the ribs anteriorly, the muscle posteriorly, and the clavicle superiorly, leading to activation of the coagulation cascade [6]. PSS may be asymptomatic, but common features include an erythematous, swollen, heavy, and painful arm, usually 24 h after the inciting event and occasionally with low-grade fever.

Doppler ultrasound is usually about 80–100 % sensitive in detecting PSS. In patients with negative Doppler ultrasound results but a high index of suspicion for PSS, computed tomography and magnetic resonance venography are useful tools for diagnosis [2, 5]; however, these modalities could be technically difficult to use, especially in an already swollen arm.

Oral anticoagulation alone for at least 6 months has been the mainstay of treatment for UEDVT; however, this has been found to be insufficient in patients with PSS. Fifteen percent of such patients end up with pulmonary embolism, and 40–90 % develop chronic disability of the affected hand [5]. CDT has a higher success rate, especially if performed within 10 days of diagnosis. This explains why early and prompt diagnosis of this syndrome is very important [6]. Contraindications for CDT are active bleeding, neurosurgery within the preceding 2 months, hemorrhagic stroke, hypersensitivity to the thrombolytic agent, and any surgery within the preceding 10 days. Thrombolysis alone clears only the thrombus. Concomitant thoracic outlet decompression is undertaken to remove the underlying cause, reduce the risk of recurrent thrombosis, and prevent long-term morbidity. This is performed by a transaxillary first-rib resection [3].

The two modalities of treatment were compared in 117 patients diagnosed with PSS [7]. In that study, Taylor and colleagues concluded that patients treated surgically had better outcomes than those treated with CDT alone. In another study, Urschel and coworkers [8] reported the largest series of patients with PSS, including a total of 626 patients treated according to their protocols, and confirmed that early thrombolysis and early surgery get better results with a lower recurrence rate and a lower rate of postthrombotic syndrome. Multimodal treatment that involves combination of all the treatment modalities also reportedly leads to better outcomes [6, 9].

In a publication by the University of California, Los Angeles [10], angioplasty and stenting were suggested instead of surgery for the treatment of PSS. This option was deemed insufficient without rib resection. If rib resection is not performed, there is a tendency for stent fracture and further dissemination of clots to occur when patients resume their activities; however, only in cases where there is a persistent narrowing of the vein after rib resection is angioplasty alone deemed sufficient.

## Conclusions

We found out that PSS remains largely a condition with which many clinicians are unfamiliar and that the tendency for many patients to leave the hospital undiagnosed is high. The following are the primary takeaway lessons of the present report:

- Given that PSS is commoner in young people who are engaged in competitive sports, upper arm swelling in such patients should raise a suspicion of PSS. If the clinician is unsure, the opinion of the vascular surgical team should be sought, as they are more familiar with the disease.
- Thompson and colleagues [11] suggested that all doctors working in general practice, emergency medicine, DVT clinics, sports medicine, and rheumatology should be aware of PSS to ensure that an early diagnosis is made so that prompt treatment can be initiated for a better overall outcome.
- More reports on PSS at all levels are encouraged.

## Consent

Written informed consent was obtained from the patient for publication of this case report and any accompanying images. A copy of the written consent is available for review by the Editor-in-Chief of this journal.

## Abbreviations

CDT: catheter-directed thrombolysis
DVT: deep vein thrombosis
GP: general practitioner
PSS: Paget-Schroetter syndrome
UEDVT: upper extremity deep vein thrombosis
